# Salt stress and salt shock differently affect DNA methylation in salt-responsive genes in sugar beet and its wild, halophytic ancestor

**DOI:** 10.1371/journal.pone.0251675

**Published:** 2021-05-27

**Authors:** Monika Skorupa, Joanna Szczepanek, Justyna Mazur, Krzysztof Domagalski, Andrzej Tretyn, Jarosław Tyburski

**Affiliations:** 1 Centre for Modern Interdisciplinary Technologies, Nicolaus Copernicus University, Toruń, Poland; 2 Chair of Plant Physiology and Biotechnology, Faculty of Biological and Veterinary Sciences, Nicolaus Copernicus University, Toruń, Poland; 3 Department of Immunology, Faculty of Biological and Veterinary Sciences, Nicolaus Copernicus University, Toruń, Poland; CSIR - Institute of Himalayan Bioresource Technology, India, INDIA

## Abstract

Here we determined the impact of salt shock and salt stress on the level of DNA methylation in selected CpG islands localized in promoters or first exons of sixteen salt-responsive genes in beets. Two subspecies differing in salt tolerance were subjected for analysis, a moderately salt-tolerant sugar beet *Beta vulgaris ssp*. *vulgaris* cv. Huzar and a halophytic beet, *Beta vulgaris ssp*. *maritima*. The CpG island methylation status was determined. All target sequences were hyper- or hypomethylated under salt shock and/or salt stress in one or both beet subspecies. It was revealed that the genomic regions analyzed were highly methylated in both, the salt treated plants and untreated controls. Methylation of the target sequences changed in a salt-dependent manner, being affected by either one or both treatments. Under both shock and stress, the hypomethylation was a predominant response in sugar beet. In *Beta vulgaris ssp*. *maritima*, the hypermethylation occurred with higher frequency than hypomethylation, especially under salt stress and in the promoter-located CpG sites. Conversely, the hypomethylation of the promoter-located CpG sites predominated in sugar beet plants subjected to salt stress. This findings suggest that DNA methylation may be involved in salt-tolerance and transcriptomic response to salinity in beets.

## Introduction

Salinity is one of the most destructive abiotic stresses which severely affects agricultural productivity. At present more than 20% of all irrigated, arable lands is estimated as affected by soil salinity, worldwide. This problem is increasing with the excessive use of irrigation water and rapid desertification, is the latter being driven by global warming [1]. Besides genotypic differences, the way of exposure and duration of salinity determines salt stress response in plants. As proposed by Shavrukov [2], gradually increasing salt concentrations or the sustained exposure to low levels of salinity bring about salt stress. Conversely, the salt shock which is an extreme form of salt stress, occurs when plants are instantly subjected to high levels of salinity. Salt shock rarely occurs in either agricultural practice or in natural ecosystems because the salt concentration in soils usually increases gradually [2]. According to the “two phase growth response to salinity” theory, developed by Munns [3, 4], the initial effect of exposure to salinity is due to osmotic changes outside the salt-treated cells and results in the reduction in the plant’s ability to absorb water (osmotic effect). The second phase occurs due to the salt accumulation in leaves. Excessive salt concentrations in plant cells cause the enzyme’s activity inhibition and trigger the oxidative stress; ionic effect [3–5].

Sensing environmental changes and initiating gene expression response play a decisive role in plant’s survival under adverse conditions [6]. Recent studies demonstrate the importance of epigenetic modifications, such as DNA methylation, in regulating gene expression during stress response [7]. However, the molecular mechanisms linking both processes are still not clear. Furthermore, the target genes remain unidentified and the consequences of stress-induced changes in DNA methylation on the gene expression patterns are largely unknown. Further studies on the role of epigenetic modifications in plant stress response would contribute significantly to the understanding of the molecular mechanisms underlying plant response to environmental stresses. Ultimately, the results may reinforce the efficacy of breeding programs aimed on selecting new crop varieties with improved productivity and enhanced environmental stress tolerance [7, 8].

Currently, the most extensively studied epigenetic mechanism is the methylation of cytosine nucleotides to 5-methylcytosine, *i*.*e*. the DNA methylation [9]. In plants, this modification occurs in three sequence contexts: CpG, CpHpG and CpHpH (where H = A, T or C). The effect of DNA methylation on gene expression varies according to tissue type, the sequence context and the genome region *i*.*e*. whether the intergenic region or the gene body is a target for methylation. It is usually assumed that DNA methylation in the gene promoter and first exon leads to the reduction in gene expression [10, 11]. Numerous properties of the plant genome such as, its morphology, stability, differentiation, gene expression, transposable elements (TEs) transposition, chromatin structure and genome protection against mutational changes were reported to be affected by methylation [8, 9].

Sugar beet (*Beta vulgaris ssp*. *vulgaris*) is an important crop plant characterized by an elevated salt resistance. The wild ancestor of cultivated beets, *Beta vulgaris ssp*. *maritima* (sea beet) inhabits the shores of the European coast of Mediterranean Sea and Atlantic Ocean. Due to its adjustment to a highly saline environments *Beta vulgaris ssp*. *maritima* is able to withstand more severe salt stress, compared to cultivated beets [12–14].

The available reports provide a limited data on the effects of salt stress on epigenetic modifications in beets. Also the role of epigenetic factors in the differences in salinity tolerance between cultivars and subspecies within *Beta* genus has been studied to a very limited extent, so far. The single report from this field describes the role of histone acetylation in the regulation of the peroxidase-encoding gene expression under salt stress in *Beta vulgaris ssp*. *vulgaris* L. and *Beta vulgaris ssp*. *maritima* [15]. There are also two other reports on changes in DNA methylation in sugar beet. However, they do focus not on salt stress response but on bolting regulation [16, 17].

The aim of the presented research was to assess the impact of salt shock and salt stress on the level of DNA methylation in gene promoter- or first exon-located CpG islands in *Beta vulgaris ssp*. *maritima* and sugar beet. The salt shock treatment was executed by immersing the petioles of excised leaves into the salt solutions thus exposing leaves instantly to high salinity. Adding NaCl directly to the transpiration stream reduced or completely eliminated the protective effects of mechanisms regulating Na^+^ delivery to the leaves and enhanced the exposure of the photosynthetic apparatus to salinity [18–20]. For the salt stress treatment, potted plants were exposed to the gradual increments of salt concentration [20]. The salt stress and shock treatments, performed in this study, were based on ideas developed by Shavrukov [2], where the difference between the two treatments consists in the way of exposing plants to salinity. However, it should be kept in mind that alternative understanding of these terms, based on salt concentrations, instead of the duration of treatment, is also employed to describe differences in plant responses to salt treatments. Accordingly, the salt stress occurs when plants are exposed to low levels of salinity. In contrast, higher salt concentrations induce salt shock. The main component of the salt shock is the osmotic stress and the oxidative stress [21].

The methylation status of sixteen, salt-responsive genes was assessed. The relevant transcripts were up- or down-regulated under shock and/or stress either in both or one of the subspecies [20]. The CpG island methylation levels were determined using methylation-sensitive and/or a methylation-dependent restriction enzymes. Following digestion, the remaining DNA in each individual enzymatic reaction was quantified by real-time PCR using primers that flank a region of interest. We hypothesized that (i) the CpG islands in salt-responsive genes are unequally methylated in beet subspecies differing in salt tolerance, (ii) different ways of exposing plants to salinity (*i*.*e*. stress *vs* shock) provoke specific, treatment mode-dependent changes in methylation patterns in salt-responsive genes, (iii) salt-dependent changes in CpG island methylation are correlated with the salt-induced alterations in gene expression level. Furthermore, the putative relation between salt tolerance of given subspecies, salt-dependent change in CpG methylation level and gene expression was discussed.

## Materials and methods

### Plant material

*Beta vulgaris ssp*. *maritima (B*. *maritima)* and sugar beet, *Beta vulgaris* ssp. *vulgaris* cv. Huzar (*B*. *vulgaris* cv. Huzar) were used as a plant material. Taxonomic classification of plants used in the study was based on Lange et al. 1999 [22]. *B*. *maritima* seeds were obtained from National Germplasm Resources Laboratory (Beltsville, MD, USA) and the sugar beet seeds from Greater Poland Sugar Beet Breeding—WHBC Poznań (Poland). Sugar beet (*Beta vulgaris ssp*. *vulgaris*) variety Huzar was selected for the experiments, being moderately salt-tolerant, as based on the results of previous experiments [23]. The seeds were sown into pots filled with sand and vermiculite (1/1 v/v) and plants were watered regularly with half-strength Hoagland solution. Plants were cultured four weeks in a growth chamber with a photoperiod of 16 h of light and 8 h of darkness with standard irradiation of 30±5 μmol m^-2^ s^-1^, provided by T8 15 W 6500 K “Daylight” tubes (POLAMP, Poland). The temperature regime was 25°C during the day and 18°C in the night. Subsequently, treatments with salinity were performed.

### Exposing plants to salinity

#### Salt shock

Fully developed first true leaves were excised, cut to the same petiole length, and immersed into plastic vials containing 25 ml NaCl-unsupplemented, half strength Hoagland solution (salt shock control leaves) or the same medium but supplemented with 300 mM NaCl (salt shocked leaves) and kept 48 hours under photoperiod as specified above. Afterwards, the leaf samples representing entire intact leaf blades were collected for analyzes [20].

#### Salt stress

Salt treatments started when first pair of mature leaves fully developed. Over the first 16 days of treatment, plants were watered in two-day-long intervals, with half-strength Hoagland medium supplemented with increasing concentrations of NaCl, until the final concentration of 300 mM NaCl in the Hoagland medium were reached. Then the treatments with 300 mM NaCl-supplemented medium were continued for subsequent 16 days. Untreated controls were watered with NaCl-unsupplemented medium. Plants were watered with 200 ml of solution per 2 l of sand/vermiculite mixture. Leaves representing the second pair of true leaves, were collected for analysis.

Forty samples were collected, representing five biological replicates (5 leaves from five plants of one subspecies) of the single experimental setup which consisted of (i) *B*. *maritima*—salt shock-control leaves, (ii) *B*. *maritima*—300 mM NaCl salt shocked leaves, (iii) *B*. *vulgaris* cv. Huzar—salt shock-control leaves, (iv) *B*. *vulgaris* cv. Huzar—300 mM NaCl salt shocked leaves, (v) *B*. *maritima*—leaves from the salt stress-control plants, (vi) *B*. *maritima*—leaves from plants subjected to 300 mM NaCl salt stress, (vii) *B*. *vulgaris* cv. Huzar—leaves from the salt stress-control plants, (viii) *B*. *vulgaris* cv. Huzar—leaves from plants subjected to 300 mM NaCl salt stress.

### Genes analyzed in this study

Genes selected for analysis were identified in the course of previous study as changing their expression level under salt stress and/or salt shock in one or both beet subspecies (Skorupa *et al*., 2019). Sixteen genes were analyzed. The relevant genes encoded: (i) membrane aquaporin 2;1 (*Bv*PIP2;1), (ii) tonoplast aquaporin (*Bv*TIP2), (iii) mechanosensitive ion channel (MsIC), (iv) expansin (EXP), (v) EXORDIUM protein (EXD), (vi) cellulose synthase (CS), (vii) bHLH 48 transcription factor (bHLH 48), (viii) HTH-type transcriptional regulator ptxE (ptxE), (ix) ethylene-responsive transcription factor TINY (TINY), (x) peroxidase 27 (POX27), (xi) L-ascorbate oxidase encoding gene without verified chromosomal localization (AOX UN), (xii) L-ascorbate oxidase encoding gene located on chromosome 5 (AOX 5), (xiii) osmotin (OSM), (xiv) taumatin (TAU), (xv) ribosome-inactivating protein (RBP), (xvi) heat shock protein (HSP). Operationally, the analyzed genes were divided into five functional categories. Genes involved in: transmembrane transport—*Bv*PIP2;1, *Bv*TIP2, MsIC, cell wall plasticity—EXP, EXD, CS, transcriptional regulation—bHLH 48, TINY, ptxE, oxidation -reduction reactions—POX27, AOX UN, AOX 5 and response to pathogens**—**OSM, TAU, RBP, HSP.

### Selecting genomic regions for analysis and the CpG island localization

In order to detect the CpG islands associated with the aforementioned genes, the gene promoters region, defined as the genomic regions of the length of 2000 bp upstream of the transcription start site, and the first exon sequence were analyzed, using the MethPrimer 2.0 program [24]. The RefBeet-1.2.2 genome location of the studied regions and gene symbols are listed in S1 Table in S1 Appendix. Sixteen CpG islands localized in these regions were assessed for the methylation analysis. Eight islands were localized in the gene promoter regions (*Bv*PIP2;1, MsIC, EXP, CS, ptxE, POX27, AOX UN, AOX 5) and eight ones were present in the first exon of the gene of interest (*Bv*TIP2, EXD, bHLH 48, TINY, OSM, TAU, RBP, HSP). The length (nt) and %GC of the CpG islands, subjected for further analysis are presented in S1 Table in S1 Appendix. The target genomic regions were also screened for the presence of repetitive sequences: long terminal repeat (LTR) retrotransposons, Gypsy-like elements, non-LTR retrotransposons of the long interspersed nuclear element (LINE) type, short interspersed nuclear elements (SINEs). For this purpose, a web pages using the Generic Genome Browser software and data generated in the context of The Sugar Beet Genome Project, created by Heinz Himmelbauer and Juliane Dohm ware used (https://bvseq.boku.ac.at/index.shtml). The implemented algorithm was employed to blast the analyzed sequences against the reference beet genome (RefBeet-1.1) and then, to locate the repetitive sequences in the overlapping regions [25]. Selected cis-regulatory elements were identified, in the sequences, using Database of Plant Cis-acting Regulatory DNA Elements—New PLACE, while the potential sites recognized by selected salt-related transcription factors were determined using the Plant Promoter Analysis Navigator PlantPAN 2.0 program.

### Genomic DNA isolation

Genomic DNA was isolated from leaf samples using the DNeasy Plant Mini Kit (Qiagen), following the manufacturer’s protocol. Each DNA sample represented a single biological replicate. DNA quantity and quality was determined using Qubit dsDNA HS Assay Kit and Qubit fluorometer (Life Technologies). After that, the DNA integrity was checked using HS DNA Kit and Agilent 2100 Bioanalyzer (Agilent Technologies). The extracted genomic DNA was stored at -20°C for later use.

### DNA methylation analysis

DNA methylation analysis in the CpG context was based on the use of methylation-sensitive or a methylation-dependent restriction enzymes. The relative fractions of methylated and unmethylated DNA were subsequently determined by comparing the amount in each digest with that of a mock (sample with no enzymes added) digest using a ΔCT method. The assay was carried out with 1 μg genomic DNA using EpiTect II DNA Methylation Enzyme Kit (Qiagen), with minor modifications of the manufacturer’s protocol to accommodate for the study organism. Specifically, we hand designed the primers, and optimized PCR conditions for efficient amplification in consultation with the manufacturers.

Following digestion, the DNA in each individual enzyme reaction was quantified by real-time PCR using RT^2^ qPCR SYBR Green MasterMix (Qiagen). The primer sequences were designed to flank a region containing CpG island linked to investigated genes. Each primer sequence consisted of 24 nucleotides and equal percentage of G+C and A+T (S2 Table in S1 Appendix). PCRs were carried out in a total volume of 10 μl. Each reaction contained 1 μl DNA template, 1 μl each gene-specific primer (final concentration 0.3 μM), 2 μl H_2_O and 5 μl RT^2^ qPCR SYBR Green MasterMix (Qiagen). The reactions were carried out with LightCycler 480 (Roche), using the following thermal profile: 10 min at 95°C, then 55 cycles of 20 s at 95°C, 20 s at 65°C and 45 s at 72°C and a final extension for 7 min at 72°C. SYBR Green fluorescence was recorded after elongation step of each cycle. The specificity of the amplifications was checked by melting curve analysis performed by heating the samples from 70*°*C to 90*°*C temperature increments of 0.5°C with simultaneous fluorescence detection.

Before the routine assays were performed, the suitability of all primer pairs was tested by assessing the PCR efficiency. For this purpose the standard curves were constructed using serial dilutions of DNA as a template. The qPCR was performed on serially diluted samples in triplicate. The qPCR data were plotted as the fluorescence signal versus the threshold cycle number. The PCR efficiencies were calculated from the slopes of the standard curves according to the equation: E = 10^[*−*1*/*slope]^ [26].

### qPCR amplification of the genomic sequences analyzed

In order to determine the specificity and quality of the qPCR assay, the reaction efficiencies were determined for all pairs of the PCR primers used. The qPCR assays were characterized by high efficiency values, which comprised in the range from 1.85 to 1.99. It confirmed their suitability for analyzes undertaken in this study (S3 Table in S1 Appendix).

### Data analysis

The relative participation of methylated and non-methylated DNA fractions were determined according to manufacturer’s protocol using a ΔCt method (EpiTect II DNA Methylation Enzyme Kit, Qiagen). In order to determine the significance of differences in CpG methylation levels between experimental variants, a two-way analysis of variance (ANOVA, P <0.05) was performed. In order to investigate the correlation between the relative expression level of the analyzed genes and the methylation level of their CpG islands, the Pearson’s linear correlation was calculated using the SigmaPlot 11.00 software.

### Fold-change expression levels in response to salt treatments

The fold-difference in the gene expression levels in beets subjected to salt-stress or salt-shock were calculated based on results of leaf transcriptome sequencing, published by Skorupa et al. 2019 [20]. The FPKM values were used for calculating the fold-change expression ratios between salt-treated plants and respective controls—S2 Table in S1 Appendix.

## Results

### Structural analysis of the CpG island–containing, selected genomic regions

**Fig 1:**
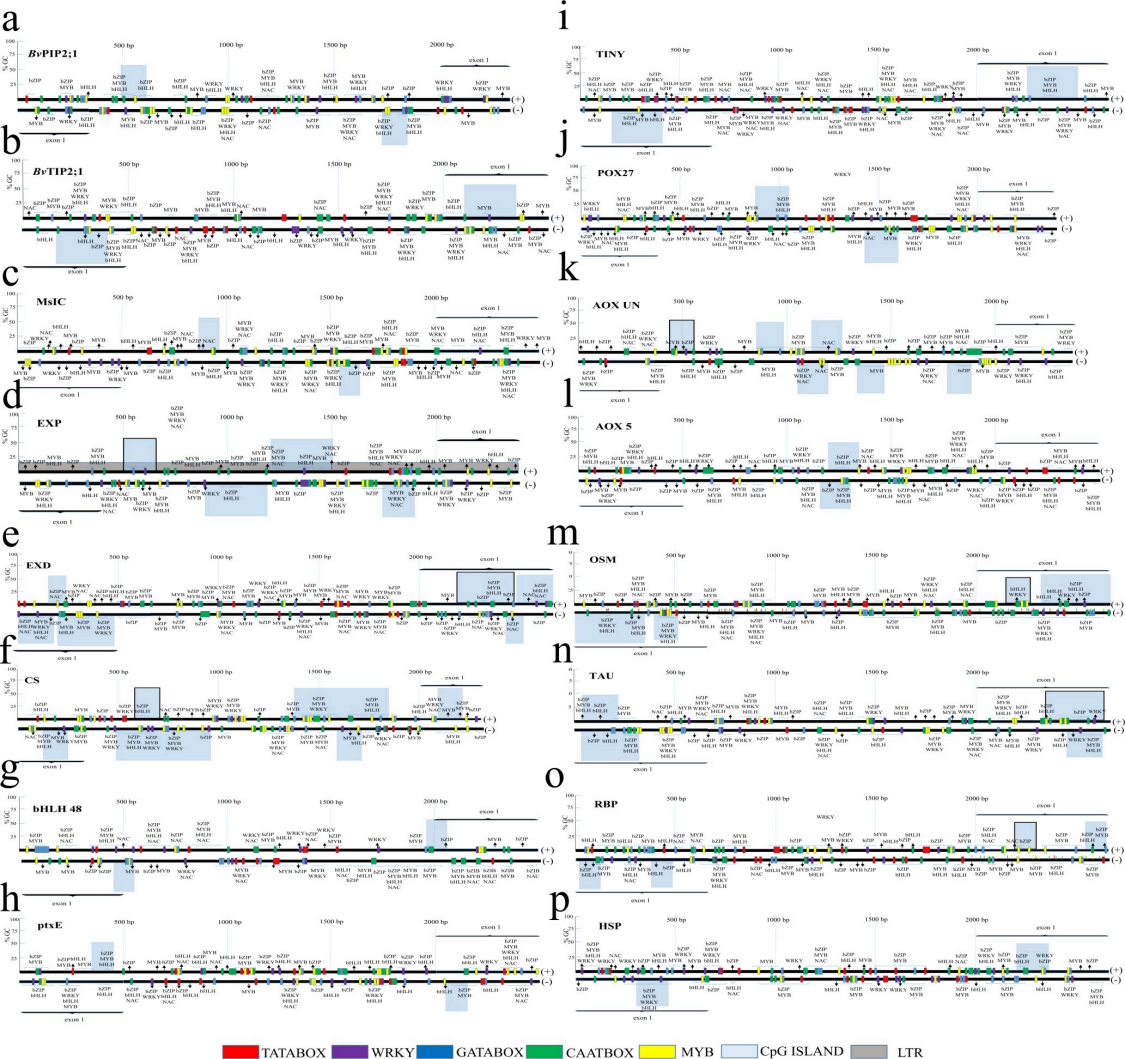
Structure and motif composition of the promoter regions of the genes analyzed in this study. The analyzes were performed on sugar beet sequences derived from the RefBeet-1.2.2 reference genome. Genes encoding: a—membrane aquaporin 2;1 (*Bv*PIP2;1), b—tonoplast aquaporin (*Bv*TIP2), c—mechanosensitive ion channel (MsIC), d—expansin (EXP), e—EXORDIUM protein (EXD), f—cellulose synthase (CS), g—bHLH 48 transcription factor (bHLH 48), h—HTH-type transcriptional regulator ptxE (ptxE), i—ethylene-responsive transcription factor TINY (TINY), j—peroxidase 27 (POX27), k—L-ascorbate oxidase encoding gene without verified chromosomal localization (AOX UN,), l—L-ascorbate oxidase encoding gene located on chromosome 5 (AOX 5), m—osmotin (OSM), n—taumatin (TAU), o—ribosome-inactivating protein (RBP), p—heat shock protein (HSP) were analyzed. CpG islands (marked as light blue squares), LTR (marked as gray squares), selected cis-regulatory sequence elements (TATABOX—red squares, CAATBOX—green squares, WRKY—purple squares, MYB—yellow squares, GATABOX—blue squares) and potential places recognized by specific stress-related transcription factors (bZIP, MYB, WRKY, bHLH, NAC) are shown. For genes with more than one CpG island, the analyzed island is marked with a black frame.

The gene promoter regions of the length of 2000 bp upstream of the transcription start site and the adjacent exon sequence were screened for the presence of CpG sites. In parallel, core promoter elements were mapped in the promoter regions. Both the gene promoter regions and the exon sequence were screened for the presence of the different cis-acting regulatory sequences. The core promoter sequences, such as TATABOX and CAATBOX were present in all gene promoters analyzed (Fig 1). The genomic regions displayed different patterns of the CpG island distribution. The gene promoter–located sites were identified in genes coding for *Bv*PIP2;1 (Fig 1A), MsIC (Fig 1C), EXP (Fig 1D), POX27 (Fig 1J), AOX UN (Fig 1K) and AOX 5 (Fig 1L). Conversely, the promoter regions of genes encoding *Bv*TIP2;1 (Fig 1B), EXD (Fig 1E), ptxE (Fig 1H), TINY (Fig 1I), OSM (Fig 1M), TAU (Fig 1N), RBP (Fig 1O) and HSP (Fig 1P) did not contain CpG sites. However, they were present in the first exon sequence. As a result of screening the target sequences for the presence of repetitive sequences, a long terminal repeat (LTR) retrotransposon was detected in the entire sequence corresponding to the EXP-encoding gene. These results indicated that the fragment identified as the CpG island of the EXP promoter region is located within the transposon sequence (Fig 1D). Other sequences, analyzed in this study, turned out to be free from TEs (Fig 1A–1C and 1E–1P).

The region related to the gene encoding CS contained the CpG islands both in the promoter and in the adjacent exon (Fig 1F). The bHLH48 corresponding region, was marked by the CpG island overlapping the promoter—first exon border (Fig 1G). Importantly, numerous sequence motives recognized by the transcription factors (TFs), involved in salt stress response (representing bZIP, MYB, WRKY, NAC and bHLH families), were identified in the regions analyzed. The putative TF binding sites were identified both, in the gene promoter regions and the adjacent exons (Fig 1).

### Overview of the effect of salinity on the CpG methylation in the selected genomic regions

Regardless of beet taxonomic classification, five CpG islands localized in **gene promoter** regions were hypermethylated, under salt shock, whereas nine ones were hypomethylated under this kind of salt treatment. Salt stress resulted in the hypermethylation of four islands. Five islands was hypomethylated under stress. If differences between the two subspecies were taken into consideration, it was revealed that shocked *B*. *maritima* displayed the hypermethylation of three islands and the same number was hypomethylated. In turn, stress treatment resulted in four islands being hypermethylated. Noteworthy, the hypomethylation was not observed in this subspecies, under stress. Salt-shocked, excised leaves of *B*. *vulgaris* cv. Huzar reacted to the same treatment with only two CpG islands hypermethylated, whereas six islands were hypomethylated. Under stress, we did not detect hypermethylation within the set of islands analyzed, but five islands underwent hypomethylation in the crop (Table 1).

**Table 1:** The number of hyper- and hypomethylated CpG islands in *B*. *maritima* and *B*. *vulgaris cv*. Huzar under salt stress and shock.

Directions of changes in methylation states of CpG islands of selected genes					
		hypermethylation		hypomethylation	
		promoter region	exon	promoter region	exon
**SALT SHOCK (both subspecies)**		**5**	**7**	**9**	**6**
**SALT STRESS (both subspecies)**		**4**	**6**	**5**	**9**
**SALT SHOCK**	***B*. *maritima***	**3**	**4**	**3**	**3**
	***B*. *vulgaris* cv. Huzar**	**2**	**3**	**6**	**3**
**SALT STRESS**	***B*. *maritima***	**4**	**3**	**0**	**5**
	***B*. *vulgaris* cv. Huzar**	**0**	**3**	**5**	**4**

In both subspecies, seven **exon-located** CpG islands were hypermethylated and six ones were hypomethylated under shock treatment. Salt stress resulted in six and nine islands being hyper- and hypomethylated, respectively. Salt-shocked leaves of *B*. *maritima* were marked by four hypermethylated CpG islands and three hypomethylated ones. Salt stress caused the hypermethylation of three islands, whereas five ones were hypomethylated. In *B*. *vulgaris* cv. Huzar the equal number of three hypermethylated islands were detected under both, stress and shock. The number of three and four islands were hypomethylated under shock and stress, respectively (Table 1).

### Analysis of the methylation level of CpG islands of individual genes with respect to taxonomic classificationand to the mode of salt treatment

**Fig 2:**
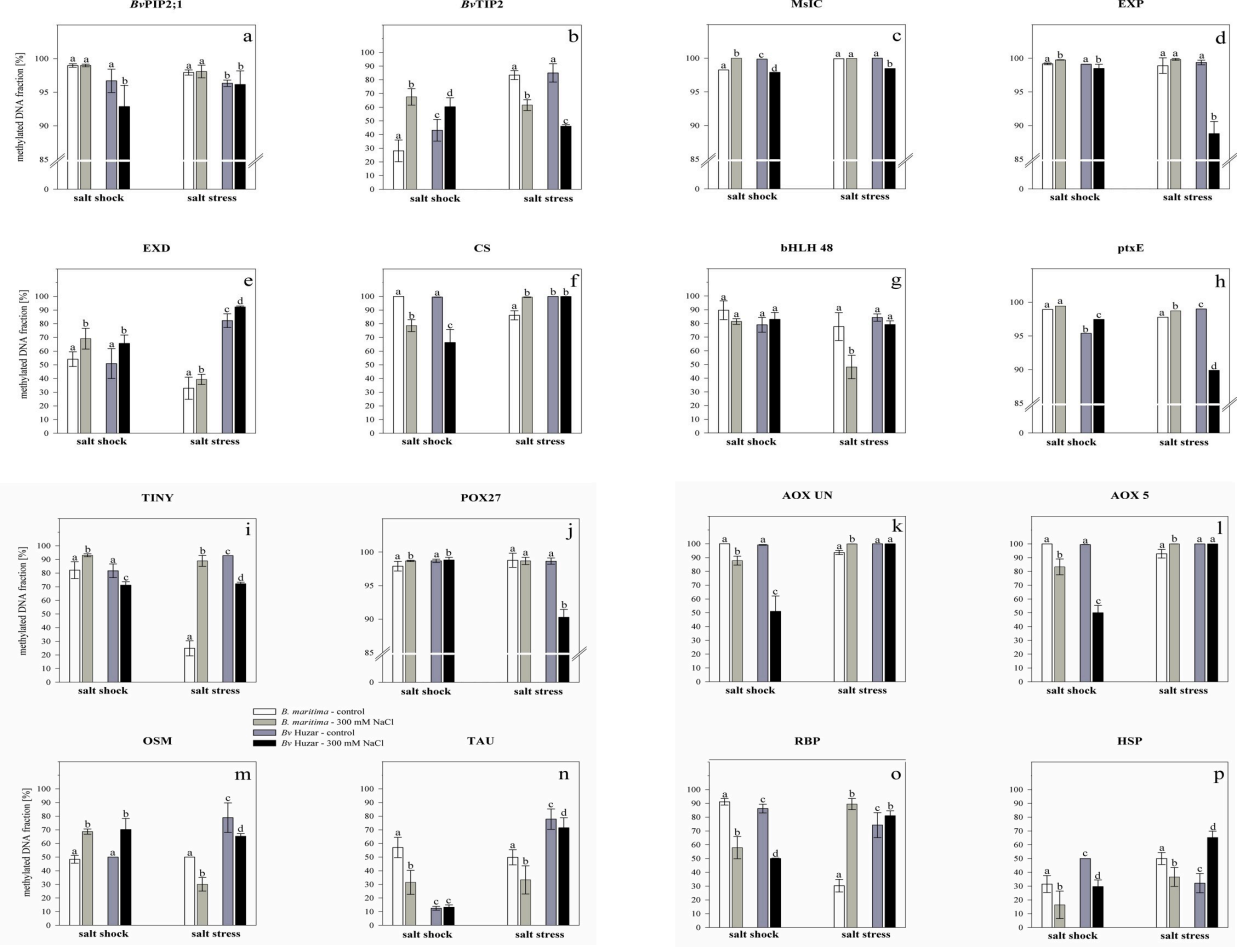
**Methylated DNA fraction [%] of CpG islands in promoter region or first exon of the genes encoding:** a—membrane aquaporin 2;1 (*Bv*PIP2;1), b—tonoplast aquaporin (*Bv*TIP2), c—mechanosensitive ion channel (MsIC), d—expansin (EXP), e—EXORDIUM protein (EXD), f—cellulose synthase (CS), g—bHLH 48 transcription factor (bHLH 48), h—HTH-type transcriptional regulator ptxE (ptxE), i—ethylene-responsive transcription factor TINY (TINY), j—peroxidase 27 (POX27), k—L-ascorbate oxidase encoding gene without verified chromosomal localization (AOX UN,), l—L-ascorbate oxidase encoding gene located on chromosome 5 (AOX 5), m—osmotin (OSM), n—taumatin (TAU), o—ribosome-inactivating protein (RBP), p—heat shock protein (HSP) determined in *B*. *maritima* and sugar beet (*B*. *vulgaris* cv. Huzar) subjected to salt shock or stress. White bars—*B*. *maritima*, control, light gray bars—*B*. *maritima*, 300 mM NaCl, dark gray bars—*B*. *vulgaris* cv. Huzar, control, black bars—*B*. *vulgaris* cv. Huzar, 300 mM NaCl. Different letters above bars indicate significant differences at p < 0.01 (ANOVA followed by Tukey’s test).

Results of the experiments showed that changes in methylation level in the CpG islands of both, the promoters and the first exon regions of the analyzed genes were affected by salinity, the method of its application, and differed between the subspecies. The results are shown in Fig 2. Four categories of salt-dependent changes of methylation patterns were identified with respect to the subspecies and the mode of the treatment:

- salinity dependent change, *i*.*e*. the same direction of change occurred under stress and shock in both subspecies. The gene coding for the EXORDIUM protein was the single representative of this category. The methylation level of CpG island located in the first exon of this gene significantly increased compared to the control, under both, stress and shock in both *B*. *vulgaris subspecies*.
- subspecies-dependent/treatment mode-independent, *i*.*e*. the same direction of change occurred under stress and shock for a given subspecies but the opposite direction of change was observed, regarding taxonomic classification. A single example falling into this category is the exon-located CpG island of the gene coding for TINY transcription factor. Its methylation pattern changed in the same direction under both stress and shock but was different between the subspecies. The percentage of methylation of this CpG island significantly increased, in *B*. *maritima*, and significantly decreased in *B*. *vulgaris* cv. Huzar, when compared to the respective controls.
- subspecies-independent/treatment mode-dependent, *i*.*e*. the opposite direction of change occurred under stress and shock but the same direction of change was observed in both subspecies. The analysis of the influence of stress and shock on the methylation level in three CpG islands revealed same directions of changes in both beet subspecies. Exon-located islands in genes encoding OSM and *Bv*TIP2 were marked by a significant increase in methylation under salt shock and decrease under salt stress. Conversely, the CpG island in gene coding for RBP displayed a significant decrease in methylation level under salt shock and an increase under salt stress.
- subspecies-dependent/treatment mode-dependent *i*.*e*. two subspecies examined displayed different directions of change in methylation patterns which were not consistent between treatments. In *B*. *maritima*, the similar methylation patterns were observed in five CpG islands located in promoter regions of genes encoding EXP, MsIC, POX27, *Bv*PIP2.1 and ptxE. Salt shock resulted in small but significant increase in the level of methylation in islands located in EXP, MsIC, whereas salt stress did not cause any changes. The methylation level of islands in *Bv*PIP2.1 and ptxE remained unchanged under shock and in the case of the former one, also under stress. Salt stress resulted in very slight but significant increase in CpG island methylation in ptxE gene promoter. Common methylation pattern, consisting in methylation decrease under shock and its increase under stress, was observed for CS, AOX UN, AOX 5 gene promoter-located islands. The exon-located islands from TAU, HSP and bHLH 48 underwent the decrease in methylation level under both, salt shock (for bHLH 48 the reduction in methylation was not significant) and stress (Fig 2).

Analysis of the results of salt treatments in *B*. *vulgaris* cv. Huzar, revealed a significant but small increase in methylation in EXP, ptxE and POX27 gene promoters under salt shock, whereas the salt stress induced a reduction in their methylation level. The methylation of CS, AOX UN and AOX 5 gene promoters decreased under shock and did not change under stress. A significant decrease in the percentage value of methylated fraction of DNA in *B*. *vulgaris* cv. Huzar occurred in exon-located CpG island of the HSP-encoding gene, whereas the salt stress caused a reverse effect. The level of methylation in exon-located CpG island of TAU significantly decreased under salt stress, but did not change after a salt shock treatment, as compared to the control. The small but significant decreases in CpG island methylation in promoters of MsIC and *Bv*PIP2;1 were detected in plants subjected to both treatments (with exception of *Bv*PIP2;1, were no effect of stress was observed). The methylation of the exon-located island in bHLH 48-encoding gene did not change under neither shock nor stress (Fig 2).

Analysis of the methylation level of CpG islands of individual genes, with respect to the mode of salt treatment, revealed the common pattern of salt shock-induced methylation changes for CS, AOX UN, AOX 5, HSP and TAU. Both subspecies were characterized by hypomethylation in aforementioned genes in shocked leaves. Under salt stress *B*. *maritima* displayed hypermethylation in CS, AOX UN and AOX 5, whereas no significant changes were detected in *B*. *vulgaris* cv. Huzar. Conversely, the methylation in *Bv*PIP2;1, EXP, MsIC, POX27 and ptxE remained unchanged in *B*. *maritima* and decreased in cv. Huzar (Fig 2).

### Differences in transcript levels

The effects of salt stress and salt shock on gene expression were shown in Table 2. Regardless of the subspecies and the type of treatment, a significant increase in the level of gene expression was observed for MsIC and HSP, and a significant decrease was revealed in the case of genes coding CS, POX27 and RBP. Significant changes in the level of transcripts representing other genes were also observed in treated plants when compared with respect to controls. However the direction of changes were inconsistent between the modes of treatment and/or beet subspecies. Under both types of treatment with salinity, the subspecies-dependent changes were observed in the case of TINY. The significant decrease in its transcript level was noted in *B*. *vulgaris* cv. Huzar, when compared to the control, whereas no changes occurred in *B*. *maritima*. An increase in expression under salt shock, and a decrease under salt stress were detected for genes encoding *Bv*PIP2;1, *Bv*TIP2, EXP, EXD, bHLH 48, ptxE, AOX UN and AOX 5. An opposite direction of changes, where significant decrease in expression occurred under salt shock and the increase under salt stress was observed for OSM and TAU.

**Table 2:** Changes in expression levels (relative to control) of analyzed transcripts responding to salt shock or stress treatments in *B*. *maritima* or *B*. *vulgaris* cv. Huzar.

	transcript ID	gene symbol	relative expression level			
			SALT SHOCK		SALT STRESS	
			MK vs M300	HK vs H300	MK vs M300	HK vs H300
1	XLOC_053207	*Bv*PIP2;1	4,54	4,63	-3,69	-2,04
2	XLOC_047607	*Bv*TIP2	7,62	11,52	-4,41	-3,36
3	XLOC_012114	MsIC	2,06	1,06	1,49	2,67
4	XLOC_054944	EXP	130,32	52,10	-1,41	-4,41
5	XLOC_016733	EXD	97,06	110,61	-7,35	-18,9
6	XLOC_044713	CS	-3,04	-2,09	-1,77	-3,96
7	XLOC_025824	bHLH 48	4,63	4,24	-1,52	-1,12
8	XLOC_038203	ptxE	4,61	4,93	-6,10	-9,35
9	XLOC_050744	TINY	1,17	-9,33	1,55	-18,97
10	XLOC_036706	POX27	-19,66	-14,5	-14,5	-15,01
11	XLOC_010202	AOX UN	11,12	22,05	-6,71	-1,84
12	XLOC_031211	AOX 5	16,49	26,29	-2,75	-1,58
13	XLOC_046291	OSM	-5,22	-1,36	5,40	4,40
14	XLOC_030805	TAU	-2,14	-12,93	6,20	11,47
15	XLOC_053015	RBP	-3,53	-2,07	-3,52	-1,83
16	XLOC_020341	HSP	1,20	2,72	2,77	4,82

Numbers in cells represent either expression fold ratio for up-regulated genes or reverse of fold ratio for down-regulated ones (denoted with a minus sign). MK—*B*. *maritima*, control, M300—*B*. *maritima*, 300 mM NaCl, HK—*B*. *vulgaris* cv. Huzar, control, H300—*B*. *vulgaris* cv. Huzar, 300 mM NaCl.

**Fig 3:**
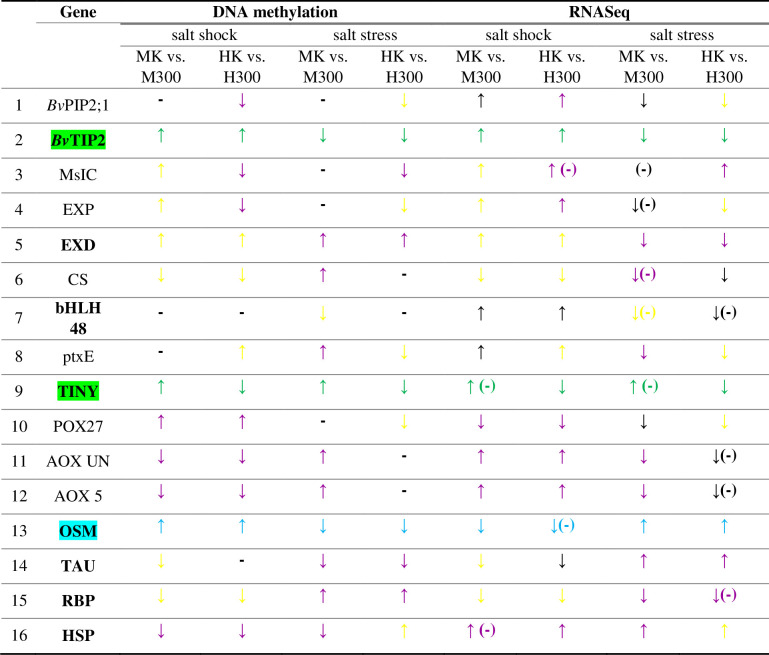
The effect of salt shock and stress on the direction of change in CpG island methylation and gene expression in *B*. *maritima* and *B*. *vulgaris cv*. Huzar. MK—*B*. *maritima*, control, M300—*B*. *maritima*, 300 mM NaCl, HK—*B*. *vulgaris cv*. Huzar, control, H300—*B*. *vulgaris cv*. Huzar, 300 mM NaCl, ↑—increase in DNA methylation or gene expression, ↓—decrease in DNA methylation or gene expression, ‘–‘—no significant changes in DNA methylation, (-)—gene expression fold-change below 2, aquamarine—opposite directions of changes in the whole experimental setup (inverse correlation), green—the same directions of changes in the whole experimental setup (positive correlation), violet—opposite directions of changes, yellow—the same directions of changes. Genes with exon-located CpG islands are highlighted, genes with promoter-located CpG island are unmarked.

In order to analyze the correlation between the levels of gene expression and methylation of the CpG islands corresponding to the genes in question, the directions of changes (increase or decrease) in these parameters were summarized in Fig 3, according to the data presented on Fig 1 and Table 2. The opposite directions of changes over the entire experimental system occurred in the case of the OSM gene. Taking only the salt shock into account, similar relation was observed in both beet subspecies in the case of POX27, AOX UN, AOX 5 and HSP and solely in *B*. *vulgaris* cv. Huzar for *Bv*PIP2;1, MsIC and EXP. Under salt stress both subspecies displayed the inverse correlation between gene expression and CpG island methylation for EXD, TAU and RBP. Moreover, the opposite directions of change in both parameters were detected specifically in *B*. *maritima* for CS, ptxE, AOX UN, AOX 5 and HSP and for MsIC in *B*. *vulgaris* cv. Huzar. The same direction of changes in CpG methylation and the gene expression was observed for *Bv*TIP2 and TINY, over the entire experimental setup, in both subspecies. The rest of experimental variants analyzed, displayed either unidirectional changes in the state of methylation and the transcript number or their methylation level did not change significantly (Fig 3).

Pearson’s correlation analysis confirmed the existence of a significant negative correlation (inverse relationship) between changes in the level of gene expression and the level of their methylation in the entire experimental system, i.e. under both treatments and in both beet subspecies for the OSM (S4 Table in S1 Appendix). Under salt shock the significant negative relationships between the two parameters were observed for the *Bv*TIP2, AOX UN, AOX 5 and HSP in both beet subspecies but for MsIC solely in *B*. *vulgaris* cv. Huzar. When analyzing the salt stress-treated plants, the direction of changes in CpG methylation status vs transcript level was significantly negative in *B*. *maritima* for the CS, ptxE, AOX UN, AOX 5, TAU, RBP, HSP and MsIC, EXD and RBP in *B*. *vulgaris* cv. Huzar (S4 Table in S1 Appendix). Positive correlation (unidirectional change) between CpG methylation status and expression level was revealed in both subspecies for the EXD, CS, ptxE, TINY and RBP, under shock and *Bv*TIP2 and TINY, under stress. Solely in *B*. *maritima*, the positive correlation was significantly for MsIC, EXP and TAU in salt shocked leaves and for bHLH48 in leaves of salt stressed plants. Under salt stress, the significant positive correlation between CpG methylation and gene expression of EXP, CS, ptxE, POX27 and HSP occurred only in *B*. *vulgaris* cv. Huzar (S4 Table in S1 Appendix).

## Discussion

The aim of our experiments was to analyze and compare the methylation level of CpG islands of the promoter regions or first exons of selected salt-responsive (both, up- and down-regulated) genes in the cultivated variety of sugar beet and its halophytic ancestor. The salt treated plants and the respective controls were subjected for analysis. Therefore, both, the salt-induced, as well as the constitutive differences in the CpG island methylation status between the studied beet subspecies were detected.

Importantly, most of the CpG islands were characterized by very high level of DNA methylation. Especially the gene promoter-located CpG island were marked by elevated methylation, exceeding 90%. These results are in line with data emphasizing that the *Beta vulgaris* genome is methylated to a very high degree. Niederhuth and co-workers (2016) analyzed DNA methylomes of 34 different angiosperm species. The results showed that from among of all plant genomes subjected to analysis, the *Beta vulgaris* genome was the most methylated one. In *Beta vulgaris*, the methylation level in the context of CpG rises up to 92.5%, whereas the relevant methylation percentage for CpHpG and CpHpH was ~ 81.2% and ~ 18.8%, respectively [27].

We demonstrated that DNA methylation levels of the majority of the CpG islands analyzed were significantly different between the two beet subspecies tested. Since the sugar beet cultivar with moderate salt tolerance capacity and the salt-tolerant, halophytic subspecies, were juxtaposed in our study, the observed variation in methylation levels may be connected to the differences in the salinity tolerance between the subspecies. Similar observations were reported by Garg et al. 2015, who examined three rice cultivars differing in the ability to tolerate salt stress or drought. The authors showed that all cultivars reacted with genome hypermethylation under stress but the drought-tolerant one was characterized by the highest DNA methylation level [28]. Wang et. al. 2015, also demonstrated significant differences in the level of DNA methylation between sensitive and salt-tolerant cotton accessions. Tolerant accessions were characterized by DNA hypermethylation under stress, whereas the hypomethylation was observe in the sensitive ones [29].

The methylation levels of the analyzed regions changed significantly under the influence of shock and stress. Undoubtedly, this fact indicates the involvement of DNA methylation processes in response to salinity and possibly also in its tolerance in beets. If both subspecies are taken together, the number of CpG islands undergoing hyper-or hypomethylation under shock and stress is similar, which means that neither increase nor decrease in the level of DNA methylation predominates. However, it should be kept in mind that the frequency of methylation marks might change over time of salt treatment, which was different for stressed and shocked plants. Therefore, due to the difference in the duration of the salt exposure, direct comparisons of DNA methylation patterns between the two models of salt treatment are not reliable. Noteworthy, the methylation status of some sequences changed under both treatments, which makes these sequences strong candidates for targets for studies on salt-affected DNA methylation.

The analysis was performed using the limited pool of sixteen genes. To address this issue comprehensively, a global evaluation of the DNA methylation of the beet genome should be carried out, to track the genome-scale effects of shock and stress. Treatments with methylation inhibitors, such as zebularine or 5-azacytidine, might also be important to estimate the role of global methylation changes in reactions to salt treatments. According to the available reports, salinity undoubtedly changes the pattern of DNA methylation in plant genomes. This finding was confirmed by studies conducted on a clonal plant *Alternanthera philoxeroides* where the variation in the methylation patterns increased under the influence of salt stress [30]. The majority of the literature reports DNA hypermethylation under salt stress. Olive tree (*Olea europaea*) cultivars were exemplary for this case showing significant increase in the level of DNA methylation caused by salinity. The variety characterized by low salinity tolerance displayed lower increase in CpG methylation level, when compared to a salinity-tolerant one [31]. Similar results were obtained by examining two wheat cultivars differing in salt tolerance. However, in this case the salt-induced increase in the level of methylation in the sensitive variety was statistically insignificant [32]. Other examples of the DNA hypermethylation under salinity were provided by studies conducted on *Phoenix dactylifera*, *Medicago truncatula* or cotton [11, 33, 34]. Experiments carried out on four rice genotypes indicated that salt-induced increase in the level of DNA methylation occurred in one of two salt-sensitive cultivars and in a highly salt-tolerant one, while a decrease was observed in the second sensitive genotype and in a moderately salt-tolerant one. These study shows that the relation between the DNA methylation pattern and the genotype’s sensitivity to salinity may not be clearly established [8]. It should be emphasized that the aforementioned studies were performed using intact plants subjected to salt stress conditions, whereas there is no literature data concerning the epigenetic modifications in the excised, salt-shocked leaves.

The subspecies differed with respect to effects of salt treatments on the CpG island methylation. Namely, under the influence of both shock and stress the decrease in the level of DNA methylation predominated in *B*. *vulgaris* cv. Huzar. In *B*. *maritima*, the hypermethylation occurred with higher frequency than hypomethylation. The difference was far more marked when only the promoter regions of salt stress-treated beets were subjected for comparison. It is assumed that gene promoter hypometyhylation usually promotes transcriptional activation [10, 11]. Therefore, this findings are in line with results of the transcriptomic study of Skorupa et al. 2019, who demonstrated that under salt stress, significantly more genes are up-regulated in sugar beet when compared to *B*. *maritima*. This suggests that the survival strategy in crop beet involves substantial transcriptional mobilization to activate the mechanisms, which are not in use under optimal conditions, but which warrant acclimation to salinity [20]. The hypomethylation in salt-responsive genes may be involved in this mechanism. However, the magnitude of the methylation/demethylation-dependent regulation of transcriptomic response to salinity may depend on several factors. Namely, the position of the CpG site within the target region, the fraction of cells that change their methylation status and the tissue-specific functions of target genes. Further studies are required to address these issues.

The genes analyzed here for their methylation patterns were identified as the salt-responsive ones in the course of the transcriptomic analysis and may potentially be involved in acquiring the salt- and other abiotic stress tolerance [20]. These genes were classified into five functional categories. Analyzing changes in the methylation level of CpG islands did not reveal common patterns within individual categories. The exception was, the “pathogen response” category, were the methylated DNA fractions of the CpG islands were consequently lower in both the control and salt-treated plants, when compared to other group of genes. Skorupa et al. 2019, indicate that a constitutive expression of the pathogen response genes occurs in *B*. *maritima*, suggesting adaptation of this subspecies to the biotic stress [20]. Low methylation of the first exon-located CpG island in “pathogen response” genes may be at least partly involved in maintaining their elevated expression level in halophytic beet. In sugar beet, these genes were expressed at lower level in untreated plants with respect to halophytic beet [20], which also corresponded with the higher level of methylation in relevant CpG island, when compared to *B*. *maritima*.

Analysis of the sequences corresponding to the gene encoding EXP showed that the presence of the GC-rich region is related to the sequence that is a part of the long terminal repeat (LTR) retrotransposon. TEs coexist with their host largely because CpG methylation suppresses their transcription [35]. There is a lot of literature data showing that the effect of stress on the plant induces changes in methylation in many regions of the TE sequence leading to their mobilization/silencing [35, 36]. Analyzes of the methylation level of the GC-rich region within TE of the EXP-encoding gene showed a slight decrease in the level of DNA methylation under salt shock conditions and a significant decrease under salt stress, compared to the control, in a *B*. *vulgaris* cv. Huzar. This suggests that the stress response in sugar beet may affect the mechanisms leading to TE mobilization. These results constitute a premise for the further research on, the largely unexplored field of the putative role of TE methylation in salt response in beets. Xu et al. (2018) demonstrated demethylation of many TE regions under water deficit stress [37]. Many other researchers demonstrated the transcriptional up-regulation of TE genes and their involvement, as local enhancers, in the regulation of the gene expression under abiotic stresses [38, 39].

The Pearson correlation analysis revealed that, for the majority of genes, the salt-treatment driven changes in the CpG island methylation and the gene expression levels were significantly correlated. The correlation between the studied parameters were either negative (inverse correlation) or positive (unidirectional changes, S4 Table in S1 Appendix). It was frequently reported that DNA hypermethylation of gene promoter and first exon regions is usually followed by decrease in gene expression, whereas the hypomethylation corresponds to transcriptional up-regulation i.e. the negative correlation occurs [11, 40–42]. Regarding the entire experimental setup (i.e. involving both beet subspecies and both treatments), the aforementioned pattern turned out to be significant only for OSM. Taking only the **salt shock** into account, similar finding was detected in both beet subspecies in the case of POX27, AOX UN, AOX 5 and HSP and solely in *B*. *vulgaris* cv. Huzar for *Bv*PIP2;1, MsIC and EXP. Under **salt stress** both beet subspecies displayed significant the inverse correlation between gene expression and CpG island methylation for EXD, TAU and RBP. Moreover, the opposite directions of change in both parameters were revealed specifically in *B*. *maritima* for CS, ptxE, AOX UN, AOX 5 and HSP and for MsIC in *B*. *vulgaris* cv. Huzar. It should be emphasized that the large group of the aforementioned genes code for apoplast proteins involved in the regulation of cell wall plasticity and cell wall building. The POX27, AOX UN, AOX 5 encode the enzymes involved in the apoplast redox metabolism and the regulation of the redox-mediated cross-linking between the cell wall polymers. The CS is a cellulose synthesizing enzyme involved in the synthesis and deposition of new wall polymers. The EXP as a protein with a cell wall loosening activity, also fall into the first category. On the other hand, genes coding for *Bv*PIP2;1, MsIC may participate in regulation of the turgor–driven cell expansion by mediating transmembrane water and ion transport. Since the cell expansion and turgor regulation is strongly dependent on the cell wall properties, the DNA-methylation-dependent mechanism, regulating the expression of genes involved in the cell wall dynamics and transmembrane transport may be important for regulating cellular expansion under salinity. The same possibly applies to the “pathogen response genes”, *i*.*e*. EXD, TAU, HSP and RBP. However, functional linking the proteins from this category to salt-response requires further studies. It should be emphasized that under stress, the inverse correlation between gene expression and CpG island methylation was observed for the cell wall dynamics-related genes, solely in *B*. *maritima* but not it sugar beet. Whether this finding is functionally relevant to the contrasting tolerance properties of the two beet subspecies will have to be addressed in the course of further studies.

The positive correlation between CpG methylation and the gene expression, implicating that both parameters change in the same direction, was observed for *Bv*TIP2 and TINY, over the entire experimental setup, in both subspecies. For the rest of genes, the unidirectional pattern of changes in the state of methylation and the transcript number was not consistent over the experimental setup (for example EXD, CS, RBP in *B*. *maritima*), or their methylation level did not change significantly. In the latter cases, the results did not reveal a consistent relation between the level of methylation and expression of genes differentially expressed under salinity, in our experimental system. Similar to our results, the deviations from “methylation/expression inverse correlation” were reported in numerous scientific reports. The analyzes carried out on *Medicago truncatula* [11], rice [8, 28], apple [37] or *Arabidopsis thaliana* [43] may serve as an example.

If the inverse correlation between DNA methylation of CpG islands and the transcripts level cannot be established, transcriptional alternations can be explained by involvement of other epigenetic factors which may directly or indirectly affect gene expression and/or modify the effects of DNA methylation [11, 28]. Especially, histone modifications, which may either repress or promote gene expression, also play a critical role in both plant development and plant responses to stress. At the epigenetic level, histone acetylation, methylation and phosphorylation have been reported to influence gene expression under salt stress response [44–49]. Usually, H3 methylation at lysine 4 (H3K4) and 36 (H3K36) are associated with gene activation, whereas H3 deacetylation, H3K9 methylation and H3K27 methylation are linked to transcriptional repression [49–51]. The relationship between histone modifications, the activity of enzymes involved in these processes and salt stress tolerance was demonstrated in maize [52], *Arabidopsis thaliana* [53] and rice [54, 55]. Changes in DNA methylation in the other two sequence contexts (CpHpG and CpHpH) should also be taken into account, which may also affect the expression level of the genes tested.

Furthermore, the *in silico* analysis revealed the presence of multiple, putative binding sites for the TFs in the promoter regions or first exons of selected genes (Figs 1 and 2). The role of many of these TFs in the regulation of gene expression under salinity is well recognized. Functional studies would be required to verify the role of the sequence motives, identified in the studied regions. However, it is possible that different mechanisms (i.e. TF-dependent mechanisms and epigenetic-dependent ones) overlap, and possibly interact, regulating the activity promoters of genes, analyzed in this study. For example, the target sequences for DNA methylation within the promoter may not be engaged in the transcription factor binding. It has also been proven that DNA methylation can block the binding of a transcription factor only if the specific sites are affected [56]. In summary, it cannot be concluded that the rule of the inverse correlation between DNA methylation and transcriptional response is universally obeyed during salt stress in plants.

## Conclusion

In conclusion, salinity affects the level of CpG island methylation in gene promoters and first exons of salt-responsive genes in sugar beet and its halophytic ancestor, *Beta vulgaris ssp*. *maritima*. Methylation status of several target sequences were affected by both treatments performed in the study, i.e. the salt stress and the salt shock. This finding indicates the involvement of DNA methylation processes in response to salinity and its tolerance in beets. Salt tolerant halophytic beet displayed gene promoter hypermethylation with higher frequency, when exposed to salt stress, which points to transcriptional gene silencing as a component of salt response in this subspecies. Conversely, the gene promoter hypomethylation was a predominant response to this kind of salt treatment in sugar beet. Consecutively, transcriptional mobilization, involved in salt acclimation strategy in this plant, may be promoted. Comparing the effects of salinity on DNA methylation level and the expression of selected genes revealed that CpG island hypermethylation resulting from salt treatment was not always followed by transcriptional down-regulation and vice versa. Deeper insight into the complexity of the interaction between DNA methylation and gene expression may allow to identify new aspects of the response to and tolerance of salinity in species with different sensibility to this type of stress. Understanding the role of epigenetic modifications in response to abiotic stress in plants can have a significant impact on breeding crops with increased tolerance to stress.

## Supporting information

S1 Appendix (PDF)
