# Supplementary material for: Salt stress and salt shock differently affect DNA methylation in salt-responsive genes in sugar beet and its wild, halophytic ancestor
Source: PLoS One. 2021 May 27;16(5):e0251675. doi: 10.1371/journal.pone.0251675 (PMC8158878; doi:10.1371/journal.pone.0251675)
Supplement: S1 Appendix — (PDF) [file pone.0251675.s001.pdf]

Monika Skorupa[1,2*¶], Joanna Szczepanek[1&], Justyna Mazur[1&], Krzysztof Domagalski[1,3&], Andrzej Tretyn[1,2&], Jarosław Tyburski[1,2¶]

Salt stress and salt shock differently affect DNA methylation in salt-responsive genes in sugar beet and its wild, halophytic ancestor

[1]Centre for Modern Interdisciplinary Technologies, Nicolaus Copernicus University, Toruń, Poland

[2]Chair of Plant Physiology and Biotechnology, Faculty of Biological and Veterinary Sciences, Nicolaus Copernicus University, Toruń, Poland

[3]Department of Immunology, Faculty of Biological and Veterinary Sciences, Nicolaus Copernicus University, Toruń, Poland

¶ These authors contributed equally to this work.

& These authors also contributed equally to this work.

Author for correspondence:
monika_skorupa@umk.pl
Tel: +48 56 665 60 80
ORCID 0000-0003-1868-5370

S1 Table. The genomic locations of the genomic regions and the CpG islands analyzed. Genes with exon-located CpG islands are highlighted, genes with promoter-located CpG island are unmarked.

**gene symbol**	**genome location (NCBI - RefBeet-1.2.2)**	**NCBI gene symbol**	**length of CpG island [bp]**	**%CG of CpG island**
*Bv*PIP2;1	NC_025820.2:16167440-16169755	LOC104903241	133	55
***Bv*TIP2**	NW_017567469.1:c536991-534515	LOC104908845	214	55
MsIC	NW_017567640.1:c87959-83819	LOC104884687	102	55
EXP	NC_025817.2:c3181432131814204	LOC104903841	221	51
**EXD**	NC_025812.2:c11260032-11256798	LOC104892013	552	53
CS	NC_025818.2:c21517813-21515522	LOC104899148	132	58
**bHLH 48**	NC_025814.2:19606816-19609508	LOC104889251	169	50
**TINY**	NC_025819.2:36704029-36708026	LOC104902271	257	52
ptxE	NC_025817.2:c14908059-14905283	LOC104895991	131	55
POX27	NC_025817.2:c2441819-2439428	LOC104895045	168	57
AOX UN	NW_017567596.1:c166256-163877	LOC104884409	103	53
AOX 5	NC_025816.2:c3584274-3581714	LOC104892300	150	52
**OSM**	NC_025818.2:41731165-41734174	LOC104900142	126	52
**TAU**	NC_025816.2:3727984-3730952	LOC104892311	122	55
**RBP**	NC_025819.2:c13731212-13728030	LOC104903129	244	51
**HSP**	NC_025813.2:c2649314-2646420	LOC104905072	105	51

S2 Table. Primer sequences and the lengths of PCR products.

	**gene symbol**	**primers sequence 5′->3′**		**length of the PCR product [bp]**
1	*Bv*PIP2;1	F	TGTGAACAGCCATCCCTGAACATG	434
		R	GTGACGACTTCAATTGTGATGGCC	
2	*Bv*TIP2	F	CTCCGTGAAGAGGAGACTAGAAAC	337
		R	ATGTCCACCAGAGATGTTAGCTCC	
3	MsIC	F	AGTAAACGCCTGCATGCCATAGTG	354
		R	TTTGGGTTGTGTTAGTTGGGGTCG	
4	EXP	F	GCTACATCGGGTTACAAGGTTGTG	320
		R	CCCACTCGAACAATACACCCTTTG	
5	EXD	F	AAGAAACACCCCACTAGAAGACCC	655
		R	AAACGGGTTAGTCACAGTAGCAGC	
6	CS	F	AGTAAGTGTTGGCCCAGTGGTGAT	255
		R	CTAGGATTTTAAGTGCGGGGTAGC	
7	bHLH 48	F	ACCGAGATTCGATCCGTGAACTAC	299
		R	TTAAACTCCGGCGAATCTTCCGTC	
8	ptxE	F	ATGCAGTTTCCGGTCCAACATGTC	370
		R	ATGTAGGAGTGTGCACCAGTGTGA	
9	TINY	F	GCGTCAACCACAATTGATCAGCAC	240
		R	TGGTACTGGTGCTGTTGCTGTTAG	
10	POX27	F	TCATAGGCTTCTCCTCAACTTCCG	256
		R	TGGATACCTCAACCTAGTAGAGGC	
11	AOX UN	F	CTCCTTCTCACTCCCAATAACCAC	105
		R	AAGTCGCACATTAGCTGACCATGC	
12	AOX 5	F	ATCATAACCCTAGACATGGCCACG	179
		R	TAGTGAGCCAAACATGAACCGGTG	
13	OSM	F	TAATCCTTACCTTAGCCCAAGCCC	172

		R	TGCGTGTTTGGGACAGGAAATGAG	
14	TAU	F	AACCAAACAATCCTACCGACACCC	167
		R	TGGTCTTCCACCTTTGTGGGCATT	
15	RBP	F	GGCTCAACACCAGTCACACTTAAG	318
		R	TTTGAAGTCTCCACTCTCCACCAC	
16	HSP	F	CCGACCAACAATGTCACTAATCCC	187
		R	TTTCCAGTCAATTCGTGCGTTGGC	

S3 Table. The qPCR efficiency values for the genes analyzed.

	Gene	Efficiency		Gene	Efficiency
1	***Bv*PIP 2;1**	1,875	9	**TINY**	1,935
2	***Bv*TIP2**	1,867	10	**POX27**	1,853
3	**MsIC**	1,943	11	**AOX UN**	1,900
4	**EXP**	1,971	12	**AOX 5**	1,944
5	**EXD**	1,991	13	**OSM**	1,932
6	**SC**	1,932	14	**TAU**	1,994
7	**bHLH 48**	1,943	15	**RBP**	1,967
8	**ptxE**	1,903	16	**HSP**	1,874

S4 Table. Correlation between the relative expression level of the analyzed genes and the methylation level of their CpG islands based on the Pearson's linear correlation. Asterisk stands for statistical significance (if p <0.05).

Gene	SALT SHOCK				SALT STRESS			
	B. maritima		***B. vulgaris* cv. Huzar**		***B. maritima***		***B. vulgaris* cv. Huzar**	
	correlation coefficient (r)	p-value	correlation coefficient (r)	p-value	correlation coefficient (r)	p-value	correlation coefficient (r)	p-value
***Bv*PIP2;1**	0,218	0,604	-0,655	0,078	-0,011	0,980	0,327	0,429
***Bv*TIP2**	-0,878	0,004*	-0,936	0,001*	0,873	0,005*	0,878	0,004*
**MsIC**	0,757	0,030*	-0,873	0,005*	0,652	0,079	-0,873	0,005*
**EXP**	0,873	0,005*	-0,689	0,059	-0,671	0,069	0,878	0,004*
**EXD**	0,764	0,027*	0,764	0,027*	-0,549	0,159	-0,883	0,006*
**CS**	0,873	0,005*	0,873	0,005*	-0,873	0,005*	0,878	0,004*
**bHLH 48**	-0,546	0,162	0,436	0,280	0,878	0,004*	0,663	0,073
**ptxE**	0,764	0,027*	0,878	0,004*	-0,873	0,008*	0,883	0,004*
**TINY**	0,878	0,004*	0,873	0,005*	0,878	0,004*	0,936	0,001*
**POX27**	-0,699	0,054	0,109	0,797	-0,102	0,811	0,873	0,005*
**AOX UN**	-0,873	0,005*	-0,873	0,005*	-0,873	0,005*	-0,109	0,797
**AOX 5**	-0,878	0,004*	-0,894	0,003*	-0,873	0,005*	-0,109	0,797
**OSM**	-0,900	0,002*	-0,930	0,001*	-0,936	0,001*	-0,546	0,002*
**TAU**	0,878	0,004*	-0,439	0,276	-0,768	0,026*	-0,220	0,601
**RBP**	0,873	0,005*	0,894	0,003*	-0,873	0,005*	-0,655	0,078
**HSP**	-0,719	0,044*	-0,930	0,001*	-0,783	0,022*	0,878	0,004*
